# Editorial: Immune senescence: a key driver of aging and age-related disorders

**DOI:** 10.3389/fragi.2026.1776990

**Published:** 2026-01-13

**Authors:** Jing Luan, Bryan Wu, Xingchun Gao

**Affiliations:** 1 Clinical Research Center, The 924th Hospital of the Chinese People’s Liberation Army Joint Logistic Support Force, Guilin, Guangxi, China; 2 Institute of Basic and Translational Medicine, Xi’an Medical University, Xi’an, Shaanxi, China; 3 Roche (United States), Pleasanton, CA, United States; 4 Institute of Basic Medical Sciences, School of Basic Medical Science, Xi’an Medical University, Xi’an, Shaanxi, China

**Keywords:** aging, biomarker, circadian, Crohn’s disease, immune cell, immune senescence

Aging is a multifaceted biological process characterized by progressive declines in physiological function, increasing susceptibility to diseases such as cancer, infections, autoimmune disorders, and metabolic syndromes (Ajoolabady et al., 2024; Guo et al., 2022). A growing body of evidence underscores the critical role of immunosenescence—the age-related functional deterioration of the immune system—in mediating these vulnerabilities. Immunosenescence disrupts immune surveillance, diminishes vaccine efficacy, elevates cancer risk, and fosters a state of chronic, low-grade inflammation termed “inflammaging” (Doherty et al., 2025; Franck et al., 2025; Liu et al., 2023). While traditionally viewed as an inevitable aspect of chronological aging, accumulating data reveal significant interindividual variability in immunosenescence trajectories, shaped by genetic predisposition, lifestyle factors, and environmental exposures (Wang et al., 2022). This heterogeneity underscores the urgent need to identify reliable biomarkers of immune aging and clarify the mechanisms linking immunosenescence to systemic aging and specific pathologies. Accordingly, these efforts—elucidating these mechanisms and tracking the process via biomarkers—are pivotal for developing targeted strategies to modulate immune function, ultimately mitigating age-related decline and extending health span (Ross et al., 2024).

These four articles within this Research Topic collectively enhance our understanding of immunosenescence, emphasizing its extensive impact on health and disease. Ge et al. reveal significant heterogeneity in lymphocyte subsets between infants and older adults, demonstrating that infants possess higher absolute counts of naïve and memory T cells, while older adults exhibit increased levels of NK cells. Furthermore, the age-related polarization of the CD4+/CD8+ ratio emerges as a promising biomarker for immunosenescence, offering critical insights to inform age-specific vaccination strategies and immune risk stratification in elderly populations. This shift not only reflects the progressive decline in naive T-cell reserves but also highlights the accumulation of terminally differentiated lymphocytes, which together underscore the systemic dysregulation characteristic of aging immune systems. Building on this, Zhang et al. establish a direct connection between cellular senescence and the pathogenesis of Crohn’s disease (CD), identifying senescence-related gene signatures and key hub genes such as STAT3, IL6, and IL1A that contribute to immune dysregulation and intestinal pathology. By elucidating senescence-related gene signatures that delineate molecular subtypes and correlate with immune dysregulation, their research effectively bridges fundamental aging biology with complex chronic inflammatory disorders. Lu et al. demonstrate the clinical utility of a simple systemic immune-inflammation index (SII), derived from routine blood counts, as a robust predictor of all-cause and cancer-specific mortality in older adults (≥60 years). This work underscores the translational potential of simple, inflammation-associated metrics in stratifying mortality risk. Finally, Trebing et al. address a vital methodological consideration in immune aging research by investigating the stability of a composite immune age metric (IMMAX) across the circadian cycle. Their findings validate IMMAX as a reliable biomarker while highlighting the need for standardized sampling timing in immune aging studies. Together, these studies affirm that immunosenescence is not merely a consequence of aging but an active contributor to its pathology, influencing disease susceptibility, progression, and outcomes across diverse contexts.

In summary, the convergence of evidence from these studies solidifies immunosenescence as a fundamental aspect of aging biology. The dynamic interplay between cellular senescence, inflammatory pathways, and circadian rhythms elucidated here presents novel opportunities for biomarker development and therapeutic targeting. Future research should prioritize longitudinal studies to unravel causal relationships between immune aging and systemic decline while leveraging omics technologies to identify precision interventions. By harnessing insights from immunosenescence, we can aspire not only to extend healthspan but also to redefine aging as a modifiable process—ultimately transforming our approach to age-related diseases.

## References

[B1] Ajoolabady A. Pratico D. Tang D. Zhou S. Franceschi C. Ren J. (2024). Immunosenescence and inflammaging: mechanisms and role in diseases. Ageing Res. Rev. 101, 102540. 10.1016/j.arr.2024.102540 39395575

[B2] Doherty T. M. Weinberger B. Didierlaurent A. Lambert P. H. (2025). Age-related changes in the immune system and challenges for the development of age-specific vaccines. Ann. Med. 57, 2477300. 10.1080/07853890.2025.2477300 40110678 PMC11926906

[B3] Franck M. Tanner K. T. Tennyson R. L. Daunizeau C. Ferrucci L. Bandinelli S. (2025). Nonuniversality of inflammaging across human populations. Nat. Aging 5, 1471–1480. 10.1038/s43587-025-00888-0 40588649

[B4] Guo J. Huang X. Dou L. Yan M. Shen T. Tang W. (2022). Aging and aging-related diseases: from molecular mechanisms to interventions and treatments. Signal Transduct. Target Ther. 7 (7), 391. 10.1038/s41392-022-01251-0 36522308 PMC9755275

[B5] Liu Z. Liang Q. Ren Y. Guo C. Ge X. Wang L. (2023). Immunosenescence: molecular mechanisms and diseases. Signal Transduct. Target Ther. 8 (8), 200. 10.1038/s41392-023-01451-2 37179335 PMC10182360

[B6] Ross J. B. Myers L. M. Noh J. J. Collins M. M. Carmody A. B. Messer R. J. (2024). Depleting myeloid-biased haematopoietic stem cells rejuvenates aged immunity. Nat. Apr 628, 162–170. 10.1038/s41586-024-07238-x 38538791 PMC11870232

[B7] Wang Y. Dong C. Han Y. Gu Z. Sun C. (2022). Immunosenescence, aging and successful aging. Front. Immunol. 13, 942796. 10.3389/fimmu.2022.942796 35983061 PMC9379926

